# The metabolic chronic disease prevention program in Saint Kitts and Nevis: a dietary recall study

**DOI:** 10.3389/fnut.2025.1617389

**Published:** 2025-08-08

**Authors:** Jin-Han Yang, Latoya Matthew-Duncan, Hazel Laws, Sheneil Isles, Yi-Fang Shu, Yi Lee, Lu-Xiang Lin, Shu-Yong Kang, Hsueh-Wen Hsu, Chii-Min Hwu, Hsiang-Chi Chen, Guan-Yu Su

**Affiliations:** ^1^Division of Endocrinology and Metabolism, Department of Internal Medicine, Taipei Veterans General Hospital, Taipei, Taiwan; ^2^Ministry of Health, Basseterre, Saint Kitts and Nevis; ^3^Department of Dietetics and Nutrition, Taipei Veterans General Hospital, Taipei, Taiwan; ^4^Taiwan International Cooperation and Development Fund (TaiwanICDF), Taipei, Taiwan; ^5^School of Medicine, National Yang Ming Chiao Tung University, Taipei, Taiwan

**Keywords:** small island developing states, non-communicable diseases, dietary intake, nutritional imbalance, physical activity

## Abstract

**Background:**

The Federation of Saint Christopher and Nevis faces a growing prevalence of non-communicable diseases (NCDs) due to dietary transitions, sedentary lifestyles, and unhealthy behaviors. This study aims to assess food and nutrient intake, nutrient deficiencies, and health behaviors among residents of the country.

**Methods:**

The study was conducted from February to March 2023. Individuals who lived in Saint Kitts and Nevis for at least 6 months, aged more than 18 years were randomly selected from 14 administrative parishes based on the population ratio. A 24-h dietary recall method was used to collect food and nutrient intake data. Demographic, physical activity, food intake frequency and other health behaviors were also collected. Descriptive statistical analysis was conducted on the baseline characteristics and nutritional intake, and logistic regression was used to assess the effects of demographic characteristics on unhealthy behaviors. Statistical analysis was performed using SPSS.

**Results:**

A total of 212 individuals (156 from Saint Kitts and 56 from Nevis) underwent the survey. The median BMI was 28.0, with 52.8% reporting no physical activity in the past week. The median caloric intake was 1737.8 kcal/day, with males consuming more than females. The proportional of total daily caloric intake was 46.3, 16.9 and 34.3% for carbohydrate, protein, and lipid, respectively. Other nutrients intake data revealed suboptimal calcium and vitamin A consumption, with 565.7 mg/day for calcium and 223.2 μg/day for vitamin A. The median sodium intake was 2914.9 mg, while potassium intake was 2044.3 mg. A sodium-to-potassium ratio was 1.43, which was above the WHO’s recommended value.

**Conclusion:**

In the study, we found the population of Saint Kitts and Nevis exhibits nutritional imbalance and low physical activities. These findings highlight the need for interventions to improve nutrition and promote healthy behaviors to deal with the rising burden of NCDs in the region.

## Introduction

The Federation of Saint Christopher and Nevis, located in the Caribbean, consists of two islands: Saint Kitts and Nevis. The country is divided into 14 parishes, with 9 on Saint Kitts and 5 on Nevis. As of 2023, the population of the country is 46,578 (1). The population is primarily composed of African descent (92.5%), followed by mixed-race individuals (3%), White people (2.1%), East Indians (1.5%), and others or unspecified (0.9%) (2).

Small Island Developing States (SIDS) are a group of 39 states that are geographically diverse and widely dispersed, with most located in the Caribbean and Pacific (3). Due to their high vulnerability to climate change and limited landmass, the SIDS rely heavily on food imports. More than two-thirds of Caribbean SIDS import over 60% of the food they consume, and about half import more than 80% (4, 5). Economic and trade liberalization in the SIDS has led to lower food prices. As a consequence, these countries have undergone nutritional and dietary transitions, shifting away from traditional, domestic staples, fruits and vegetables towards diets that high in processed foods, sugar, fat, salt and animal protein (6). Rapid changes in social structures and urban population growth have also contributed to sedentary lifestyles in these countries. Approximately 90.5% of females and 58.9% of males in the SIDS have low physical activity levels that do not meet WHO’s minimum recommendations (7). The changes in dietary intake and sedentary lifestyles have led to an increased prevalence of obesity and chronic non-communicable diseases (NCDs), including diabetes, hypertension, cardiovascular disease, and cancers (8).

The NCDs have replaced malnutrition and infectious diseases as the primary public health concerns. In Caribbean region, it is reported that the proportion of all deaths due to NCDs was 71.3%, and the proportion of premature deaths due to NCDs (<70 years of age) was 43.6% (9). The cause of death in Caribbean countries were 13–25% for heart diseases, 8–25% for cancer, 4–21% for diabetes, and 1–13% for cerebrovascular disease (10).

Surveys conducted in 12 Caribbean Community (CARICOM) countries show that 20 to 50% of the population has hypertension, and the prevalence of diabetes in SIDS of the Caribbean is twice the global rate, with 10 to 25% of adults affected by the disease (11). The obesity rate in adults is higher than the global average, ranging from 28.8 to 38.8% (4). Unhealthy lifestyles (including an imbalanced diet, irregular exercise, smoking, alcohol consumption, and substance abuse) are risk factors for metabolic chronic diseases (12). Furthermore, a high ultra-processed food consumption was found to be associated with chronic kidney disease, cardiometabolic disease, gastrointestinal diseases, cancers and increased mortality (13, 14). To promote nutritional health in the Federation of Saint Christopher and Nevis, it is necessary to regularly collect and monitor the food consumption status in the area. The dietary and nutrient intake data can help the government more effectively design and implement the national nutrition policies.

Therefore, in the study, we aimed to assess food and nutrient intake, the status of specific nutrient deficiencies, and dietary patterns across different age groups in the Federation of Saint Christopher and Nevis. Additionally, we also investigated the health behaviors of this population.

## Materials and methods

Individuals of the citizens of the Saint Kitts and Nevis for at least 6 months and were 18–65 years old were randomly selected from 14 administrative parishes based on the population ratio. A multi-sampling design was chosen based on geographical location, distribution of households and persons within each of the districts in Saint Kitts and Nevis to ensure that a representative sample of individuals and households in Saint Kitts and Nevis was selected to participate in the study. Those unable or unwilling to participate in the survey were excluded from the study. The study was conducted from February to March 2023.

The self-reported respondents baseline characteristics, including age, sex, height, weight, ethnicity, marital status, educational level, employment status, monthly income, physical activity level, alcohol use, and medication use, were collected. The nutrition survey team was formed by the Nutrition Department of the Ministry of Health of Saint Kitts and Nevis. The team visited each parish and registered eligible households for participation. Personnel with prior experience in nutrition surveys were recruited as survey enumerators, including those involved in the National Individual Food Consumption Survey: Saint Kitts and Nevis (2021), a collaboration between the Ministry of Health, the Food and Agriculture Organization (FAO), and the University of the West Indies. Enumerators underwent training prior to the surveys conducted by the Ministry’s nutrition department and the project dietitians. The project was continuously supervised to ensure proper case registration and monitoring. The study was approved by the Ministry of Health of Saint Kitts and Nevis, the Interim Ethics Review Committee (IERC) under approval code IERC-2022-09-058. Informed consent was obtained from respondents who agreed to participate in the survey. All participants remained anonymous, and their data were kept confidential.

The 24-h dietary recall method was administered by trained enumerators (15). Participants were asked to recall and describe every item of food and beverage consumed over exactly 24 h. The information was gathered through a systematic process of repeatedly asking open-ended questions. When necessary, the participants were asked for more detailed information such as the preparation method and type. The source and portion size of food and beverage were also recorded, and a photobook was used to help respondents identify portion sizes. The manual of food portion quantification of Saint Kitts and Nevis was developed for assessing the food consumption of individuals along with the 24-h dietary recall method (16). The collected data were recorded by the interviewers on the online platform ASA24® (Automated Self-Administered 24-h Dietary Assessment Tool). The 24-h reminder was made on a common day of the week. ASA24® is a free, web-based survey tool developed by the National Cancer Institute (NCI) in collaboration with Westat, a social science research company. It’s a globally recognized tool for food records (17). Food frequency was assessed by using a food frequency questionnaire, which is a common method to measure specific dietary behaviors and the intake of particular food groups (18). Nutritional intake was categorized into carbohydrates, proteins, lipids, alcohol and micronutrients. Carbohydrates was further subcategorized into fiber and total sugars, while micronutrients included the vitamins and minerals.

A body mass index (BMI) equal to or greater than 25 was considered overweight and a BMI equal to or greater than 30 was considered obesity. Underweight was defined as BMI less than 18.5 (19). In the study, the definitions of vegetable, fruit, and beverage intake frequency were referred to the design of previous questionnaires (18): low intake was defined as consuming 1–3 times per month; moderate intake as consuming 1–4 times per week; and high intake as consuming 5–6 times per week or daily. The frequency of exercise was defined as low if performed 0–2 times per week, moderate if 3–4 times per week and high if 5–6 times per week.

Descriptive statistical analysis was conducted on the baseline characteristics. Logistic regression was used to assess the effects of demographic characteristics on physical activity, vegetables, fruits, and beverage intake. The results were presented as hazard ratios (HRs) and 95% confidence interval. Variables with *p* value < 0.10 in univariable analysis were included in multivariable analysis. Statistical significance was defined as a *p* value < 0.05. Daily caloric and dietary intake were presented as the median, interquartile ranges, 5^th^ and 95^th^ percentage. Statistical analyses were performed using IBM SPSS Statistics software (Version 25.0, IBM Corp., Armonk, NY, USA).

## Results

There were a total of 212 respondents, with 156 from Saint Kitts and 56 from Nevis. Among the respondents of the nutritional intake survey, 94.3% were of African descent. More than half were single. Over 80% had completed secondary education and were employed full-time or part-time. The median BMI of the respondents was 28.0. The proportion of overweight and obesity was 33.0 and 37.7%, respectively (Table 1; Supplementary Table S1). To account for potential under-reporting of BMI in the sensitivity analysis, the actual BMI was estimated as 1.1 times the self-reported value (20). After adjustment, 1.4% of individuals were underweight, 14.6% had a normal BMI, 26.9% were overweight, and 57.1% were obese.

**Table 1:** Distribution of demographic characteristics of respondents stratified by island and sex.

Demographic characteristics	Saint Kitts			Nevis		
	Total (%)	Male (%)	Female (%)	Total (%)	Male (%)	Female (%)
Ethnicity						
African	150 (96.2)	68 (94.4)	82 (97.6)	50 (89.3)	19 (86.4)	31 (91.2)
Other^a^	6 (3.8)	4 (5.6)	2 (2.4)	6 (10.7)	3 (13.6)	3 (8.8)
Marital status						
Single	125 (80.6)	57 (79.1)	69 (82.1)	36 (64.3)	13 (59.1)	23 (67.6)
Not single	30 (19.4)	15 (20.9)	15 (17.9)	20 (35.7)	9 (40.9)	11 (32.4)
Education						
Master’s degree	10 (6.4)	4 (5.6)	6 (7.1)	1 (1.8)	0 (0.0)	1 (2.9)
College/university	45 (28.8)	23 (31.9)	22 (26.2)	18 (32.1)	7 (31.8)	11 (32.4)
Middle school	75 (48.1)	32 (44.4)	43 (51.2)	28 (50.0)	11 (50.0)	17 (50.0)
Elementary school	11 (7.1)	4 (5.6)	7 (8.3)	7 (12.5)	4 (18.2)	3 (8.8)
Vocational school	14 (9.0)	8 (11.1)	6 (7.1)	1 (1.8)	0 (0.0)	1 (2.9)
Not educated	1 (0.6)	1 (1.4)	0 (0.0)	1 (1.8)	0 (0.0)	1 (2.9)
Employment status						
Full-time	129 (82.7)	61 (84.7)	68 (81.0)	47 (83.9)	18 (81.8)	29 (85.3)
Part time	6 (3.8)	3 (4.2)	3 (3.6)	3 (5.4)	2 (9.1)	1 (2.9)
Retired	9 (5.8)	3 (4.2)	6 (7.1)	3 (5.4)	1 (4.5)	2 (5.9)
Student	1 (0.6)	1 (1.4)	0 (0.0)	1 (1.8)	0 (0.0)	1 (2.9)
Housewife	0 (0.0)	0 (0.0)	0 (0.0)	1 (1.8)	1 (4.5)	0 (0.0)
Unable to work	4 (2.6)	2 (2.8)	2 (2.4)	1 (1.8)	0 (0.0)	1 (2.9)
Unemployed, looking for a job	6 (3.8)	2 (2.8)	4 (4.8)	0 (0.0)	0 (0.0)	0 (0.0)
Unemployed, not planning to find a job	1 (0.6)	0 (0.0)	1 (1.2)	0 (0.0)	0 (0.0)	0 (0.0)
Monthly income (T$)^b^						
< 1,440	23 (14.7)	11 (15.3)	12 (14.3)	3 (5.4)	1 (4.5)	2 (5.9)
1,444–2,280	28 (17.9)	10 (13.9)	18 (21.4)	10 (17.9)	3 (13.6)	7 (20.6)
2,284–2,800	26 (16.7)	11 (15.3)	15 (17.9)	1 (1.8)	0 (0.0)	1 (2.9)
2,804–3,480	17 (10.9)	9 (12.5)	8 (9.5)	4 (7.1)	4 (18.2)	0 (0.0)
3,484–3,996	15 (9.6)	7 (9.7)	8 (9.5)	6 (10.7)	3 (13.6)	3 (8.8)
4,000–4,796	7 (4.5)	5 (6.9)	2 (2.4)	0 (0.0)	0 (0.0)	0 (0.0)
> 4,800	24 (15.4)	14 (19.4)	10 (11.9)	7 (12.5)	3 (13.6)	4 (11.8)
Unknown	3 (1.9)	0 (0.0)	3 (3.6)	5 (8.9)	3 (13.6)	2 (5.9)
Unwilling to disclose	13 (8.3)	5 (6.9)	8 (9.5)	20 (35.7)	5 (22.7)	15 (44.1)

a Caucasian, Indian, Hispanic and mixed-race.

b Tongan Paʻanga, usually represented as “T$.”

Among the respondents, 112 individuals reported no exercise in the past 7 days, indicating that approximately 52.8% of the population in the country may lack a regular exercise habit. The number of individuals who reported having low, moderate and high exercise was 23, 37 and 40, respectively. In univariate analysis, male engaged in physical activity 2.91 times than female, with *p* < 0.001. Those with higher income consumed more vegetables and fruits. Exercise, vegetable and fruit intake, and beverage consumption did not differ significantly by age, educational level, relationship status, or illness status (Table 2). Parameters with a *p* value < 0.1 were included in the multivariate analysis. After adjusting for income, a significant association between male sex and physical activity remained, with a hazard ratio of 2.88 (95% CI: 1.62–5.12), *p* value < 0.001 (Table 3).

**Table 2:** The effect of demographic characteristics on physical activity, vegetable, fruit, and beverage consumption in univariate logistic analysis.

Patient characteristics	Physical activity		Vegetable intake		Fruit intake		Beverage intake	
	HR (95%CI)	*p* value	HR (95%CI)	*p* value	HR (95%CI)	*p* value	HR (95%CI)	*p* value
Sex								
Female	Ref		Ref		Ref		Ref	
Male	2.91 (1.66–5.11)	<0.001*	1.01 (0.58–1.76)	0.981	0.68 (0.40–1.16)	0.159	1.56 (0.94–2.59)	0.086
Age (y)								
<25	Ref		Ref		Ref		Ref	
25–34	1.71 (0.71–4.12)	0.231	1.07 (0.38–2.96)	0.902	0.98 (0.42–2.28)	0.958	0.69 (0.31–1.52)	0.357
35–44	1.40 (0.56–3.52)	0.476	0.85 (0.29–2.43)	0.756	1.55 (0.64–3.74)	0.332	0.57 (0.25–1.31)	0.186
45–54	1.57 (0.60–4.06)	0.358	1.95 (0.67–5.64)	0.218	1.44 (0.58–3.62)	0.435	0.80 (0.33–1.90)	0.608
>55	0.80 (0.28–2.23)	0.666	1.00 (0.33–2.99)	0.993	1.49 (0.59–3.78)	0.399	0.68 (0.28–1.64)	0.396
Educational level								
Below middle school	Ref		Ref		Ref		Ref	
High school or vocational education	1.21 (0.45–3.29)	0.703	1.73 (0.61–4.92)	0.302	1.33 (0.50–3.53)	0.566	1.11 (0.46–2.67)	0.815
University or graduate school	1.33 (0.47–3.74)	0.585	2.62 (0.89–7.74)	0.080	0.76 (0.30–1.94)	0.565	1.02 (0.41–2.67)	0.971
Income (T$)^a^								
<2,280	Ref		Ref		Ref		Ref	
2,284–3,480	1.17 (0.53–2.58)	0.700	0.71 (0.31–1.66)	0.433	0.80 (0.37–1.71)	0.565	0.54 (0.27–1.08)	0.083
3,484–4,800	1.92 (0.49–3.96)	0.077	4.53 (2.13–9.64)	<0.001*	2.23 (1.09–4.53)	0.027*	1.13 (0.59–2.19)	0.713
Unwilling to disclose	1.39 (0.61–3.13)	0.432	1.20 (0.51–2.83)	0.670	2.31 (1.06–5.07)	0.036*	1.28 (0.61–2.67)	0.511
Relationship status								
Single	Ref		Ref		Ref		Ref	
Not single	1.30 (0.69–2.43)	0.415	1.48 (0.78–2.81)	0.229	1.31 (0.70–2.43)	0.397	0.89 (0.50–1.59)	0.683
Illness status								
Healthy	Ref		Ref		Ref		Ref	
Disease^b^	0.69 (0.32–1.52)	0.359	1.17 (0.55–2.47)	0.683	1.29 (0.63–2.65)	0.492	1.18 (0.59–2.33)	0.639

*p value<0.05 was considered statistically significant. CI, confidence interval.

a Tongan Paʻanga, usually represented as “T$.”

b Among the respondents, 21 were on medication for hypertension, 9 for diabetes, 5 for asthma, 1 for cancer, 1 for high cholesterol, 1 for heart disease, 1 for polio, and 1 for kidney disease, with 5 individuals having multiple conditions.

**Table 3:** The effect of demographic characteristics on physical activity in multivariate logistic analysis.

Patient characteristics	Physical activity	
	HR (95%CI)	*p* value
Sex		
Female	Ref	
Male	2.88 (1.62–5.12)	<0.001*
Income (T$)^a^		
<2,280	Ref	
2,284–3,480	1.09 (0.49–2.45)	0.835
3,484–4,800	1.66 (0.79–3.48)	0.182
Unwilling to disclose	1.60 (0.70–3.67)	0.270

*p value < 0.05 was considered statistically significant. CI, confidence interval.

a Tongan Paʻanga, usually represented as “T$.”

The amount of vegetable, fruit and beverage intake among different sexes is shown in Table 4. More individuals had high fruit intake than those with high vegetable intake, with 75 and 65 respondents, respectively. The median daily caloric intake was 1737.8 kcal (Table 5). Males had a higher median caloric intake than females. The daily carbohydrate intake for both sexes was approximately 200 g, accounting for 46.3% of total daily caloric intake. The median daily protein intake for males was 71.3 g, while females consumed a slightly higher amount at 77.7 g. The median daily lipid intake for males was 71.9 g, while for females it was 58.0 g. Males consumed 35.9% of their daily calories from fat, while females consumed 30.9%. When considering all participants together, lipid intake accounted for 34.3% of the total caloric intake. The median daily fiber intake was roughly 12 g for both sexes (Supplementary Tables S2, S3). For those with high vegetable intake, the mean fiber intake of respondents was 14.18 g (15.38 g for males and 13.22 g for females), while mean fiber intake of those with high fruit intake was 14.21 g (15.96 g for males and 13.45 g for females).

**Table 4:** The food intake of the respondents.

Food intake	Sex, *N* (%)		
	Male	Female	Total
Vegetables			
Never or low	5 (45.5)	6 (54.5)	11
Moderate	60 (44.1)	76 (55.9)	136
High	29 (44.6)	36 (55.4)	65
Fruits			
Never or low	9 (56.3)	7 (43.7)	16
Moderate	56 (46.3)	65 (53.7)	121
High	29 (38.7)	46 (61.3)	75
Beverages			
Never or low	16 (32.7)	33 (67.3)	49
Moderate	36 (46.2)	42 (53.8)	78
High	43 (50.6)	42 (49.4)	85

Low intake: consumed 1–3 times per month. Moderate intake: consumed 1–4 times per week. High intake: consumed 5–6 times per week or daily.

**Table 5:** Daily intake of calories, macronutrients, vitamins, and minerals.

Nutrients	Content				
	P5	P25	Median	P75	P95
Macronutrients					
Calories (kcal)	946.6	1277.8	1737.8	2211.0	3079.1
Protein (g)	31.8	51.6	73.3	103.2	160.4
Lipid (g)	27.9	41.5	66.3	86.6	133.9
Carbohydrate (g)	95.2	149.7	201.2	264.2	374.3
Total sugar (g)	15.8	47.4	74.8	105.9	184.2
Fiber (g)	4.2	8.4	12.0	16.9	25.4
Alcohol (g)	0.0	0.0	0.0	0.0	22.5
Vitamin					
Vitamin A (RE)^a^ (μg)	9.7	102.9	223.2	406.0	757.2
Vitamin B1 (mg)	0.5	1.0	1.4	2.1	9.4
Vitamin B2 (mg)	0.6	1.0	1.5	2.1	4.2
Vitamin B3 (mg)	9.7	16.1	24.0	34.1	67.7
Vitamin B6 (mg)	0.7	1.2	1.7	2.6	29.5
Vitamin B12 (μg)	0.6	1.8	3.5	8.0	52.9
Vitamin C (mg)	5.2	26.3	67.8	135.7	271.1
Vitamin D (μg)	0.3	1.5	3.0	6.9	36.1
Vitamin K (μg)	13.7	36.2	65.8	125.1	223.4
Folate (μg)	129.3	234.9	325.3	469.1	708.9
Minerals					
Ca (mg)	176.6	358.8	565.7	903.8	1530.2
Mg (mg)	115.3	179.2	228.0	316.8	457.5
Fe (mg)	4.8	8.4	12.0	16.6	27.4
K (mg)	936.5	1522.3	2044.3	2893.0	4459.7
Zn (mg)	3.4	5.5	8.0	11.4	26.8
Cu (mg)	0.5	0.7	1.0	1.3	2.3
Na (mg)	1268.1	2137.3	2914.9	3881.4	5630.5

a RE, retinal equivalent. P5: 5th percentile; P25: 25th percentile; P75: 75th percentile: P95: 95th percentile.

The median daily intake of vitamin A was 223.2 μg, with 228.8 μg in males and 213.7 μg in females. Males consumed vitamin C with a median of 67.0 mg, while females consumed a median of 75.3 mg. Regarding macrominerals, the median daily calcium intake was 565.7 mg (639.1 mg for males and 537.0 mg for females), the median daily potassium intake was 2044.3 mg (2054.9 mg for males and 2023.3 mg for females), and the median daily sodium intake was 2914.9 mg (2960.6 mg for males and 2900.7 mg for females). As for microminerals, the median daily iron intake for males was 12.7 mg, while the median iron intake for females was 11.7 mg. For females under 50 years old, the median daily iron intake was 12 mg. Other vitamins and mineral intake amounts stratified by age are shown in Supplementary Table S4.

## Discussion

Metabolic chronic diseases such as diabetes, hypertension, and obesity have become a burden on the healthcare system of the Federation of Saint Christopher and Nevis. The findings of this study can serve as a reference for the country in formulating dietary policies to promote the health of the population.

The results of this study show that the daily calorie intake of males was 1,801 kcal and that of females was 1,689 kcal. The daily calorie intake of males was only 70.6% of the recommended intake, while it was 88.9% in females. Overall, the collected calorie intake was low. According to the Caribbean population nutrition intake guidelines, the recommended daily caloric intake is 2,550 kcal for males and 1,900 kcal for females (21). However, considering the high prevalence of overweight and obesity among the participants, this study likely underestimated their actual daily caloric intake. Several studies have revealed the underreporting of energy intake. BMI was identified the main predictor of underreporting (22, 23), and a subsequent systematic review found that individuals with a BMI more than 30 were more likely to underreport their food intake (24). Nevertheless, 24-h dietary recalls exhibit less variation and a lower degree of underreporting compared to other dietary assessment methods (25). The observed low caloric intake, which is inconsistent with high rates of overweight and obesity in Saint Kitts and Nevis, may be explained by the social desirability biases. The phenomenon has been studied previously. Social approval tendencies are reported to be more common in females, potentially influencing self-reported dietary intake (26). We assume that our study may also be subject to this limitation.

Although caloric intake may have been underestimated, the imbalance in nutrient intake still reflects dietary issues within these populations. Compared with the Caribbean population nutrition intake guidelines (21), we found that the carbohydrate intake was lower than the recommended level of 50% or more. The proportion of energy derived from protein met the recommended 15% of total caloric intake. However, fat consumption in both sexes exceeded the recommended level of 30%, with males consuming a higher amount of fat than females. Excessive fat consumption is particularly concerning because the majority of the population engages in little or no physical activity. These findings suggest a nutritional imbalance within this population, highlighting the need for future dietary guidelines to reduce the intake of high-fat foods. Fiber intake was below the WHO’s daily recommendation of a minimum of 20 g. In terms of vitamins, vitamin A intake was below the daily recommended intake of 500 μg (females) or 600 μg (males), while vitamin B and the vitamin C met the daily recommended intake levels.

Vitamin A plays a crucial role in maintaining body functions such as vision, growth, immune function, and maintaining the integrity and survival of epithelial cells. Long-term low intake of vitamin A will lead to vitamin A deficiency (VAD), and it can cause a public health problem. Its deficiency can lead to xerophthalmia, including night blindness, corneal ulcer, and xerophthalmic fundus. This is due to poor regeneration of the visual pigment in retinal rod cells (27, 28). A low vitamin A intake during pregnancy can lead to fetal anemia, impaired immune function and increased susceptibility to infections. VAD can negatively impact hematopoietic stem cells (29). The World Health Organization (WHO) has recognized xerophthalmia as a public health problem (30). This study demonstrates that the majority of respondents’ vitamin A intake falls below the recommended threshold of 500 μg, indicating a potential risk of vitamin A deficiency within the population of this country. It is a fat-soluble vitamin that can only be obtained through diet and is primarily stored in the liver where it is not easily lost. Hence, it can be replenished by consuming foods rich in vitamin A or supplements to meet the needs for growth (31).

Calcium intake was found to be below the recommended intake levels. The study results indicate that calcium intake in the country is lower than the recommended amount of 1,000 mg. Adequate dietary calcium intake is essential for maintaining bone health and reduce the risk of osteopenia or osteoporosis (32). Postmenopausal females experience rapid calcium loss, resulting in reduced bone density and an increased risk of fractures from falls. Therefore, it is recommended that females consume adequate calcium before menopause to help prevent bone density loss (33, 34). There was also studies suggesting the association with calcium intake and lower blood pressure (35), prevention of pre-eclampsia (36), and reduced LDL cholesterol level (37). Promoting the consumption of calcium-rich natural or calcium-fortified foods, and utilizing food processing techniques that enhance calcium content, may improve calcium intake (38). Iron intake was also low, especially among premenopausal females, for whom the recommended level is 29.4 mg/day (21). Vitamin D intake was also found to be below the recommended level of 5 μg/day or 10 μg/day (for those over 50 years old). Vitamin D plays a crucial role not only in bone mineralization but also in supporting neuromuscular function. In the Caribbean region, sunlight serves as the primary source of vitamin D for most individuals (21). Therefore, individuals should be encouraged to engage in safe and adequate sun exposure to support vitamin D synthesis.

Both sexes consumed more sodium than the WHO’s recommendation of 2,000 mg/day, with males having higher sodium intake than females. On the other hand, potassium intake was found to be below the recommend levels. Overall, high sodium intake and obesity may be major factors contributing to the high prevalence of hypertension in the country. Excessive sodium intake and insufficient potassium intake are associated with hypertension and an increased risk of cardiovascular diseases (CVD) (39, 40). In recent years, the sodium-to-potassium (Na-to-K) ratio has been recognized as a more reliable indicator for assessing CVD risk and CVD-related mortality than considering sodium or potassium intake alone (41, 42). The WHO recommends limiting sodium intake to less than 2,000 mg/day and ensuring potassium intake exceeds 3,510 mg/day to maintain cardiovascular health, and the ideal Na-to-K ratio would be ≤ 1.0 (43, 44). In this study, the median sodium intake in the country was 2914.9 mg, while potassium intake was 2044.3 mg, resulting in a sodium-to-potassium ratio of approximately 1.43, which is above the WHO’s recommended value. The high hypertension prevalence was noted in the area. In the Eastern Caribbean, the prevalence of hypertension rises from 34.3% at age 40 to 82.9% in individuals aged over 70 years (45). In Saint Kitts and Nevis, the age-standardized prevalence of hypertension among adults aged 30–79 years was 45%, exceeding the global average. Among those diagnosed with hypertension, 49% received treatment, but only 22% achieved adequate blood pressure control (46). Therefore, our findings highlight the importance of adjusting electrolyte consumption in the country. Furthermore, the health behavior questionnaire indicated that more than half of the population in the country does not engage in regular exercise. Given these risk factors, including high blood pressure, a high sodium-to-potassium ratio, and a lack of regular physical activity, the risk of cardiovascular diseases and even death among the population is likely elevated.

There were limitations of the study. First, we observed a low reported caloric intake, which contradicts the high prevalence of overweight and obesity. A possible explanation is that participants may have provided inaccurate information to present themselves in a favorable light and conform to societal expectations. Second, the study investigated the individuals’ diet by recall method, which may introduce a recall bias and discrepancies with the actual intake. Future efforts should prioritize systematic evaluation and monitoring to improve the accuracy of dietary intake assessments in Saint Kitts and Nevis. Third, self-reported height and weight may be inaccurate. Several studies have investigated this issue, showing that males tend to overreport their height, while females tend to underreport their weight (47). The high BMI of the respondents may have reported biases due to self-esteem issues or social pressure (48). In other study conduct in Jamaica, females had higher actual mean BMI than males (49). However, despite potential discrepancies between self-reported and measured BMI, a high prevalence of obesity was still observed in our study. Moreover, the identified nutritional intake imbalances underscore the need for dietary improvements at the national level. Finally, a formal sample size calculation was not performed due to resource limitations, as recruitment was relied on voluntary participation through clinics and community outreach. While the final sample of 212 participants—over 0.5% of the adult population aged 18–65 (estimated at 33,000)—was proportionally drawn from all 14 parishes, the absence of a power analysis, the relatively small sample size, and the short data collection period may restrict the generalizability of our findings.

In conclusion, we evaluated the nutrition intake of the residents of the Federation of Saint Christopher and Nevis. We identified the types of nutrient deficiencies and imbalances prevalent among the population of the country. By clearly identifying nutrients that are under- or overconsumed, more targeted guidance can be provided for future nutritional recommendations. Specifically, fat and sodium intake should be reduced, while intake of potassium, calcium, iron, and vitamins A and D should be increased. The study findings support the Ministry of Health of Saint Kitts and Nevis in policy formulation and offer guidance for developing dietary programs to enhance population health.

## Data Availability

The datasets presented in this article are not readily available because the data supporting the findings of this study are not publicly available due to confidentiality agreements. Requests to access the datasets should be directed to G-YS, gysu@vghtpe.gov.tw.
