# Supplementary material for: The metabolic chronic disease prevention program in Saint Kitts and Nevis: a dietary recall study
Source: Front Nutr. 2025 Aug 8;12:1617389. doi: 10.3389/fnut.2025.1617389 (PMC12370524; doi:10.3389/fnut.2025.1617389)
Supplement: Supplementary file 1 [file Table_1.docx]

Supplementary Material

Supplementary Table S1.
The height, weight, and BMI stratified by islands, sexes, and age.

|  | Total | Island | | Sex | | Age (y) | |
| --- | --- | --- | --- | --- | --- | --- | --- |
|  |  | Saint Kitts | Nevis | Male | Female | 18-50 | >50 |
| Height (cm) | 167.6  (162.5-175.2) | 167.6  (162.6-175.2) | 167.6  (162.5-173.3) | 175.3  (168.3-182.9) | 162.6  (160.0-167.6) | 167.6  (162.6-175.3) | 166.4  (162.6-172.7) |
| Weight (kg) | 81.0  (69.1-91.4) | 81.0  (69.8-90.2) | 78.8  (67.8-92.8) | 83.3  (72.0-90.0) | 78.3  (67.2-91.8) | 81.3  (69.6-90.9) | 75.6  (67.7-91.8) |
| BMI (kg/m^2^) | 28.0  (24.2-31.3) | 28.1  (24.6-31.0) | 27.4  (23.9-32.2) | 26.2  (22.9-28.9) | 29.5  (25.5-33.1) | 28.1  (24.2-31.4) | 26.6  (24.2-30.4) |
| Underweight | 1.9% | 1.9% | 2.2% | 3.2% | 0.8% | 2.5% | 0.0% |
| Normal | 27.4% | 26.9% | 31.1% | 34.5% | 22.0% | 26.9% | 29.4% |
| Overweight | 33.0% | 35.3% | 26.7% | 37.6% | 29.7% | 31.9% | 37.3% |
| Obesity | 37.7% | 35.9% | 40% | 24.7% | 47.5% | 38.7% | 33.3% |

The height, weight and BMI are presented as median and interquartile range (IQR).

BMI: body mass index.

Supplementary Table S2.
Daily intake of energy, macronutrients, vitamins, and minerals in male respondents.

|  | Content | | | | |
| --- | --- | --- | --- | --- | --- |
| Nutrients | P5 | P25 | Median | P75 | P95 |
| Macronutrients |  |  |  |  |  |
| Calories (kcal) | 938.1 | 1330.8 | 1801.3 | 2421.3 | 2941.7 |
| Protein (g) | 30.9 | 52.3 | 71.3 | 103.3 | 152.4 |
| Lipid (g) | 28.6 | 53.7 | 71.9 | 94.4 | 125.7 |
| Carbohydrate (g) | 87.6 | 150.9 | 204.3 | 285.2 | 377.5 |
| Total sugar (g) | 21.0 | 50.9 | 79.1 | 110.2 | 166.9 |
| Fiber (g) | 4.3 | 8.4 | 11.7 | 16.7 | 29.6 |
| Alcohol (g) | 0.0 | 0.0 | 0.0 | 0.0 | 21.3 |
| Vitamin |  |  |  |  |  |
| Vitamin A (RE) ^1^ (μg) | 17.8 | 86.5 | 228.8 | 394.3 | 765.4 |
| Vitamin B1 (mg) | 0.5 | 1.1 | 1.4 | 2.0 | 3.3 |
| Vitamin B2 (mg) | 0.6 | 0.9 | 1.5 | 2.0 | 3.2 |
| Vitamin B3 (mg) | 10.0 | 16.0 | 22.4 | 29.8 | 51.2 |
| Vitamin B6 (mg) | 0.7 | 1.2 | 1.7 | 2.4 | 5.6 |
| Vitamin B12 (μg) | 0.7 | 1.8 | 3.3 | 7.5 | 12.4 |
| Vitamin C (mg) | 5.5 | 23.7 | 67.0 | 118.5 | 273.2 |
| Vitamin D (μg) | 0.3 | 1.5 | 3.2 | 7.8 | 32.0 |
| Vitamin K (μg) | 12.9 | 36.8 | 66.2 | 127.5 | 237.2 |
| Folate (μg) | 115.0 | 231.6 | 326.8 | 428.5 | 605.8 |
| Minerals |  |  |  |  |  |
| Ca (mg) | 179.2 | 405.6 | 639.1 | 944.5 | 1760.8 |
| Mg (mg) | 121.2 | 165.8 | 220.5 | 326.5 | 462.6 |
| Fe (mg) | 4.4 | 8.5 | 12.7 | 17.3 | 26.8 |
| K (mg) | 968.2 | 1521.4 | 2054.9 | 2720.5 | 4820.7 |
| Zn (mg) | 3.4 | 5.6 | 8.4 | 11.3 | 25.0 |
| Cu (mg) | 0.4 | 0.7 | 0.9 | 1.3 | 2.2 |
| Na (mg) | 1076.1 | 2271.8 | 2960.6 | 3978.4 | 5946.1 |

^1^ RE, retinal equivalent.
P5: 5^th^ percentile; P25: 25^th^ percentile; P75: 75^th^ percentile; P95: 95^th^ percentile.

Supplementary Table S3.
Daily intake of energy, macronutrients, vitamins, and minerals in female respondents.

|  | Content | | | | |
| --- | --- | --- | --- | --- | --- |
| Nutrients | P5 | P25 | Median | P75 | P95 |
| Macronutrients |  |  |  |  |  |
| Calories (kcal) | 969.8 | 1265.7 | 1689.4 | 2135.0 | 3114.9 |
| Protein (g) | 35.0 | 51.2 | 77.7 | 102.4 | 164.2 |
| Lipid (g) | 27.7 | 37.8 | 58.0 | 79.8 | 151.1 |
| Carbohydrate (g) | 105.5 | 149.3 | 201.2 | 256.8 | 354.6 |
| Total sugar (g) | 13.1 | 44.6 | 72.8 | 104.4 | 184.8 |
| Fiber (g) | 4.3 | 8.6 | 12.1 | 17.8 | 23.6 |
| Alcohol (g) | 0 | 0 | 0 | 0 | 23.8 |
| Vitamin |  |  |  |  |  |
| Vitamin A (RE) ^1^ (μg) | 9.7 | 107.8 | 213.7 | 423.8 | 730.3 |
| Vitamin B1 (mg) | 0.5 | 1.0 | 1.4 | 2.3 | 51.1 |
| Vitamin B2 (mg) | 0.6 | 1.1 | 1.5 | 2.1 | 32.4 |
| Vitamin B3 (mg) | 10.2 | 17.3 | 25.4 | 37.6 | 74.5 |
| Vitamin B6 (mg) | 0.7 | 1.2 | 1.8 | 3.2 | 51.6 |
| Vitamin B12 (μg) | 0.6 | 1.9 | 3.8 | 8.9 | 54.2 |
| Vitamin C (mg) | 5.5 | 28.5 | 75.3 | 143.1 | 243.7 |
| Vitamin D (μg) | 0.2 | 1.5 | 2.9 | 6.3 | 37.7 |
| Vitamin K (μg) | 14.6 | 36.0 | 65.8 | 114.1 | 206.9 |
| Folate (μg) | 159.4 | 235.6 | 319.0 | 507.1 | 770.3 |
| Minerals |  |  |  |  |  |
| Ca (mg) | 172.9 | 357.4 | 537.0 | 878.1 | 1437.6 |
| Mg (mg) | 113.0 | 185.6 | 229.6 | 309.1 | 448.9 |
| Fe (mg) | 5.2 | 8.4 | 11.7 | 16.0 | 27.7 |
| K (mg) | 931.7 | 1525.3 | 2023.3 | 3028.9 | 4193.1 |
| Zn (mg) | 3.4 | 5.3 | 7.7 | 11.7 | 27.7 |
| Cu (mg) | 0.5 | 0.7 | 1.0 | 1.2 | 2.5 |
| Na (mg) | 1341.1 | 2104.7 | 2900.7 | 3859.6 | 5121.0 |

^1^ RE, retinal equivalent.
P5: 5^th^ percentile; P25: 25^th^ percentile; P75: 75^th^ percentile; P95: 95^th^ percentile.

Supplementary Table S4.
Daily intake of energy, macronutrients, vitamins, and minerals in respondents aged 18-50 and >50.

|  | Age (y) | | | | | | | | | |
| --- | --- | --- | --- | --- | --- | --- | --- | --- | --- | --- |
|  | 18-50 | | | | | >50 | | | | |
| Nutrients | P5 | P25 | Median | P75 | P95 | P5 | P25 | Median | P75 | P95 |
| Macronutrients |  |  |  |  |  |  |  |  |  |  |
| Calories (kcal) | 969.8 | 1275.4 | 1743.4 | 2216.5 | 2941.7 | 913.4 | 1320.7 | 1705.0 | 2188.9 | 3111.0 |
| Protein (g) | 35.7 | 52.1 | 74.9 | 102.2 | 162.5 | 29.5 | 50.9 | 69.8 | 105.7 | 149.5 |
| Lipid (g) | 28.2 | 41.4 | 64.1 | 83.6 | 137.7 | 27.4 | 41.8 | 70.2 | 92.1 | 126.2 |
| Carbohydrate (g) | 94.8 | 146.7 | 199.3 | 264.3 | 377.5 | 100.8 | 155.1 | 209.1 | 250.2 | 350.5 |
| Total sugar (g) | 15.6 | 47.4 | 73.8 | 105.5 | 186.3 | 23.1 | 48.5 | 78.0 | 108.7 | 169.4 |
| Fiber (g) | 4.2 | 8.3 | 11.9 | 16.9 | 26.3 | 4.6 | 9.3 | 12.0 | 17.8 | 24.2 |
| Alcohol (g) | 0.0 | 0.0 | 0.0 | 0.0 | 23.9 | 0.0 | 0.0 | 0.0 | 0.0 | 17.7 |
| Vitamin |  |  |  |  |  |  |  |  |  |  |
| Vitamin A (RE) ^1^ (μg) | 9.6 | 114.8 | 229.0 | 424.0 | 860.7 | 21.9 | 70.7 | 179.4 | 367.8 | 586.6 |
| Vitamin B1 (mg) | 0.5 | 1.1 | 1.4 | 2.2 | 39.6 | 0.6 | 1.0 | 1.3 | 1.6 | 3.5 |
| Vitamin B2 (mg) | 0.5 | 1.0 | 1.5 | 2.1 | 4.5 | 0.7 | 1.0 | 1.5 | 2.1 | 3.5 |
| Vitamin B3 (mg) | 9.7 | 16.5 | 24.1 | 33.0 | 75.1 | 11.1 | 15.9 | 24.0 | 35.9 | 48.2 |
| Vitamin B6 (mg) | 0.7 | 1.2 | 1.8 | 2.7 | 51.3 | 0.9 | 1.3 | 1.6 | 2.4 | 7.5 |
| Vitamin B12 (μg) | 0.6 | 1.9 | 3.7 | 8.0 | 53.6 | 0.6 | 1.5 | 3.4 | 7.7 | 16.0 |
| Vitamin C (mg) | 5.1 | 25.7 | 67.1 | 144.8 | 295.7 | 6.2 | 39.4 | 78.0 | 115.8 | 233.3 |
| Vitamin D (μg) | 0.2 | 1.6 | 3.0 | 6.9 | 33.1 | 0.3 | 1.5 | 2.8 | 6.9 | 36.2 |
| Vitamin K (μg) | 12.5 | 36.5 | 66.5 | 121.3 | 257.7 | 17.0 | 34.6 | 60.6 | 137.6 | 204.1 |
| Folate (μg) | 131.5 | 253.1 | 334.9 | 480.7 | 709.2 | 137.9 | 222.9 | 288.1 | 466.8 | 647.3 |
| Minerals |  |  |  |  |  |  |  |  |  |  |
| Ca (mg) | 166.7 | 360.2 | 565.2 | 950.9 | 1620.5 | 195.1 | 358.3 | 565.7 | 814.9 | 1326.2 |
| Mg (mg) | 113.9 | 177.9 | 231.0 | 328.8 | 457.0 | 124.2 | 185.3 | 217.7 | 295.0 | 445.5 |
| Fe (mg) | 4.7 | 8.4 | 12.0 | 16.0 | 28.7 | 6.2 | 7.8 | 12.0 | 17.3 | 25.4 |
| K (mg) | 886.9 | 1511.3 | 2028.2 | 2969.5 | 4374.4 | 1133.8 | 1625.5 | 2087.5 | 2671.3 | 4464.0 |
| Zn (mg) | 3.4 | 5.5 | 8.3 | 11.4 | 28.4 | 3.6 | 5.5 | 7.6 | 11.3 | 18.6 |
| Cu (mg) | 0.5 | 0.7 | 1.0 | 1.3 | 2.2 | 0.5 | 0.7 | 0.9 | 1.3 | 2.5 |
| Na (mg) | 1278.0 | 2172.8 | 2992.4 | 3975.9 | 5457.9 | 1302.2 | 1933.5 | 2603.0 | 3602.6 | 5981.6 |

^1^ RE, retinal equivalent.
P5: 5^th^ percentile; P25: 25^th^ percentile; P75: 75^th^ percentile; P95: 95^th^ percentile.
